# Alcohol and Substance Use Disorders Diagnostic Criteria Changes and Innovations in ICD-11: An Overview

**DOI:** 10.32872/cpe.9539

**Published:** 2022-12-15

**Authors:** Alice Matone, Claudia Gandin, Silvia Ghirini, Emanuele Scafato

**Affiliations:** 1 Osservatorio Nazionale Alcol, Centro Nazionale Dipendenze e Doping, Istituto Superiore di Sanità, Rome, Italy; University of Zurich, Zurich, Switzerland

**Keywords:** Disease International Classification, ICD-11, substance use disorders, addictive behaviors, public health, psychoactive substances

## Abstract

**Background:**

The new revision of the ICD came into effect on January 1st, 2022, and significant changes have been introduced in the section related to substance use disorders.

**Method:**

In the present work we describe the new ICD-11 section “Disorders due to Substance Use and Addictive Behaviors” and outline the innovations in classification and diagnosis introduced, with a view to addressing the most important issues in terms of new opportunities for identifying and caring for people in need of treatment.

**Results:**

The main innovations introduced in the ICD-11 chapter of interest are the expanded classes of psychoactive substances, the introduction of single episodes of substance use, the introduction of harmful patterns of substance use and severity qualifiers for substance intoxication. Furthermore, the new category “Disorders due to addictive behaviors” has been added, including “Gambling disorder” and the new diagnostic category “Gaming disorder”.

**Conclusions:**

ICD-11 calls for renewed public health response and policies fostering the multi-professional and multidisciplinary management of alcohol and substance abuse treatment, giving to these forms of addiction new chances also towards the reaching of the UN 2030 Agenda Sustainable Development Goals.

## Highlights

- ICD-11 represents a new opportunity for those who are in need for treatment to be timely identified.
- ICD-11 allows to fill the existing therapeutic gap and increase the coverage of substance use disorders.
- ICD-11 pushes for necessary changes in the post-COVID era: an integrated approach aimed at using standard tools and training for adequate intervention.
- The new definitions adopted by ICD-11 are in line with the UN 2030 Agenda, aimed at ensuring healthy lives and promote well-being for all ages.

On January 1^st^, 2022, the 11^th^ revision of the International Classification of Disease (ICD) system, ICD-11, came into effect. The ICD is a collection of human disorders and related health conditions which is used from approximately 180 countries around the globe and is periodically revised from the World Health Organization (WHO). Disease classification and coding is crucial not only for accurate clinical diagnosis and effective communication between medical professionals, but also for epidemiological data gathering in order to monitor trends in disease prevalence and incidence, and for providing a basis for precision in research (Sanusi et al., 2022; Saunders, 2017).

Within the wide spectrum of recognized disorders that have an impact on human health and society, of non-trivial importance are disorders related to psychoactive substance use. Psychoactive substances, when taken in or administered into a person’s system, affect mental processes such as consciousness, cognition, perception, mood and emotions. Especially if untreated, substance use disorders increase morbidity and mortality risks, and can lead to major suffering and impairment in important areas of functioning, such as family, occupational and social life. Substance use disorders are associated with significant costs to society due to lost productivity, premature mortality, increased health care expenditure, and costs related to criminal justice, social welfare, and other social consequences (World Health Orgnization, 2022). Therefore, careful consideration of these spectrum of diseases within the international coding systems is necessary and unavoidable.

The section of the ICD-11 dedicated to mental health is called “Mental, Behavioral or Neurodevelopmental Disorders” (MBND), and is the result of a wide international, multidisciplinary and participative process that involved many experts and stakeholders around the World, such as mental health professionals and users of mental health services (Gaebel et al., 2020; Reed et al., 2019). The WHO Department of Mental Health and Substance Abuse (DMHSA) assigned a dedicated advisory group for the revision of ICD-10 chapter on mental health, and working groups were established worldwide in order to collaborate to the development of the new MBND chapter in ICD-11. Based on the available evidence, the working groups proposed improvements to the classification system related to mental health, resulting in a beta draft that was made available online from 2015, in order to receive additional comments and inputs (Gaebel et al., 2020). The 11th version of the ICD was approved in May 2019 by the World Health Assembly, after which the WHO DMHSA published the Clinical Description and Diagnostic Guidelines (CDDG) for the ICD-11 MBND, as the result of a multidisciplinary and international collaboration process that lasted for over a decade (Reed et al., 2019). The main criteria adopted for the development of the ICD-11 MBND process have been the consideration of clinical utility, adherence to scientific soundness, and global applicability. Furthermore, since the development of the Diagnostic and Statistical Manual of Mental Disorders (DSM)-5 was partially contemporary to the one of the ICD-11 Clinical Descriptions and Diagnostic Guidelines, the coherence between the two tool was considered of crucial importance, particularly in terms of minimizing arbitrary differences between the two (Reed et al., 2019).

The DSM is one of the most widely used diagnostic tools for mental disorders, it is published from the American Psychiatric Association (APA), and the 5^th^ revision was completed in 2013. The DSM covers all categories of mental health disorders and has a widespread importance and influence on how disorders are diagnosed, treated, and investigated. Although great efforts have been made to harmonize the ICD-11 with the DSM-5, the two systems do have some differences, also considering that they have, to some extent, different aims. While the DSM-5 aims at providing a common research and clinical language for mental health problems, the ICD-11 pays particular attention to issues of clinical utility in a broad range of settings, aiming at global applicability, and especially the area of ‘addictions’ has been handled by the latest revisions of the two systems with somewhat divergent approaches, that will be discussed later in this article (Grant & Chamberlain, 2016).

The ICD-11 MBND chapter includes disorders related to substance use in the section “Disorders due to Substance Use and Addictive Behaviors” (Saunders et al., 2019; World Health Organization, 2019). Several important changes have been made in this section with this last revision, that reflect adjustment to modern times, in terms of new substances, behaviors and psychological dynamics (Poznyak et al., 2018). In this work we describe the changes in substance use disorders and addictive behaviors classification between ICD-10 and ICD-11 and their implications, specifically:

1. expanded classes of psychoactive substances;
2. introduction of single episodes of substance use;
3. introduction of harmful patterns of substance use;
4. severity qualifiers for substance intoxication;
5. introduction of the category “Disorders due to addictive behaviors” that includes “Gambling disorder” (previously under “Habit and impulse disorders”) and the new diagnostic category “Gaming disorder”.

Globally, the need for treatment for substance use disorders did not yet reach a satisfying level and the changes introduced in the ICD-11 have important implications for public health in terms of opportunities for improved monitoring, prevention and treatment and for restructuring of health services in such a way that patient-centered care is prioritized. Interventions must be supported from informed strategies and one of the main priorities in this respect is to provide health professionals with an effective tool for identifying people in need (Poznyak et al., 2018). Therefore, with the present manuscript we aim at providing professionals with valuable insights by outlining the main changes in the 11th revision of the ICD that will have important implications in terms of public health approaches.

## ICD-11 “Disorders due to Substance Use and Addictive Behaviors”

**Figure 1:**
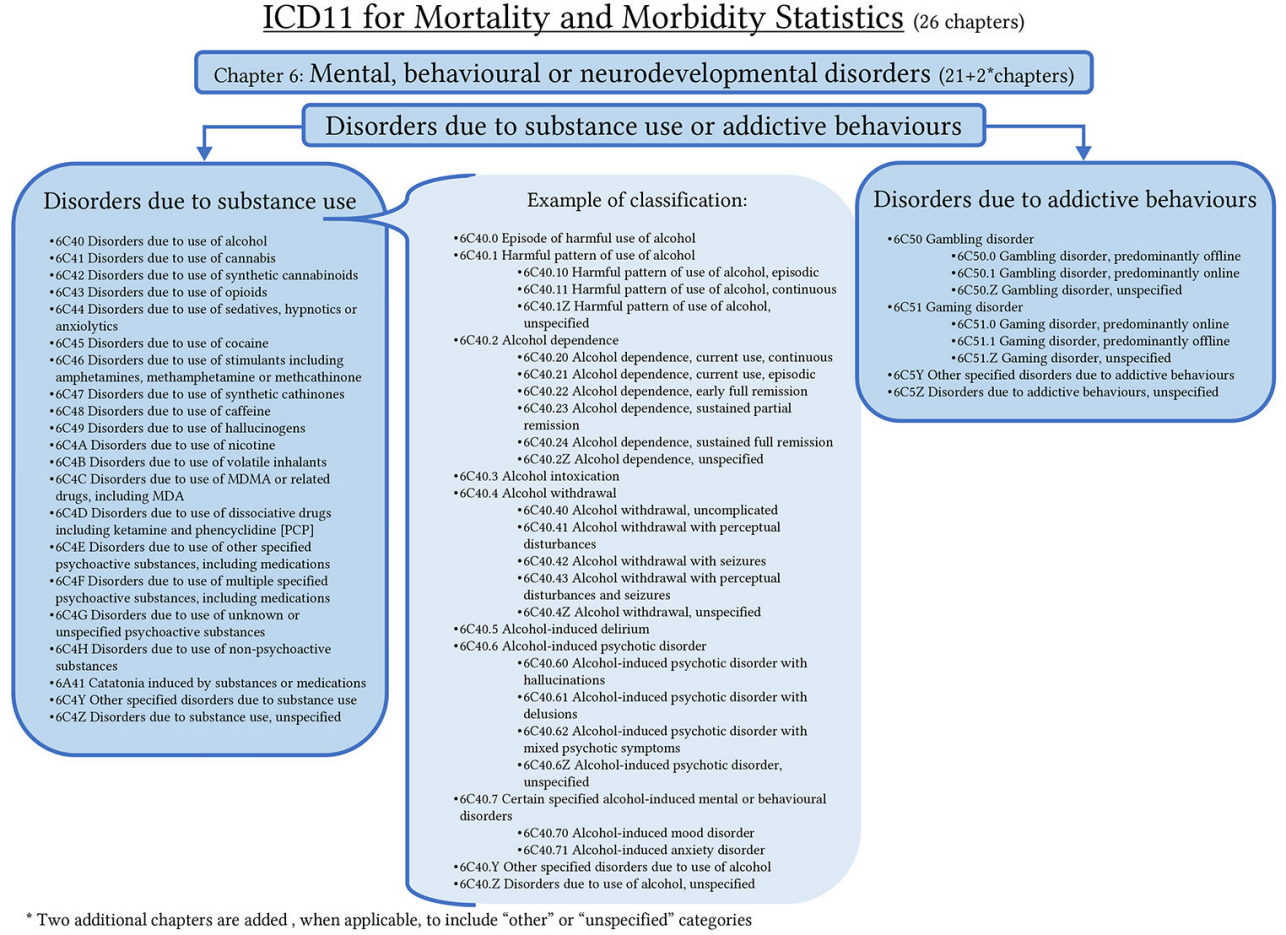
Schematic Representation of Chapter 6 of the ICD-11, “Disorders due to Substance Use and Addictive Behaviors”

Chapter 6 of the ICD-11, “Mental, behavioral and neurodevelopmental disorders”, includes a new grouping of conditions in the 12th section called “Disorders due to Substance Use and Addictive Behaviors” (see Figure 1) which is described as follows:

“*Disorders due to substance use and addictive behaviors are mental and behavioral disorders that develop as a result of the use of predominantly psychoactive substances, including medications, or specific repetitive rewarding and reinforcing behaviors*” (World Health Organization, 2019).

The WHO strategic approach to minimize harm from substance use is reflected in this new version of the ICD-11, where the public health approach to substance use and addictive behaviors is emphasized from diagnoses (Reed et al., 2019). The section is divided itself in two parts, “Disorders due to substance use” and “Disorders due to addictive behaviors”.

### Disorders Due to Substance Use

#### Expanded Classes of Psychoactive Substances in ICD-11

**Figure 2:**
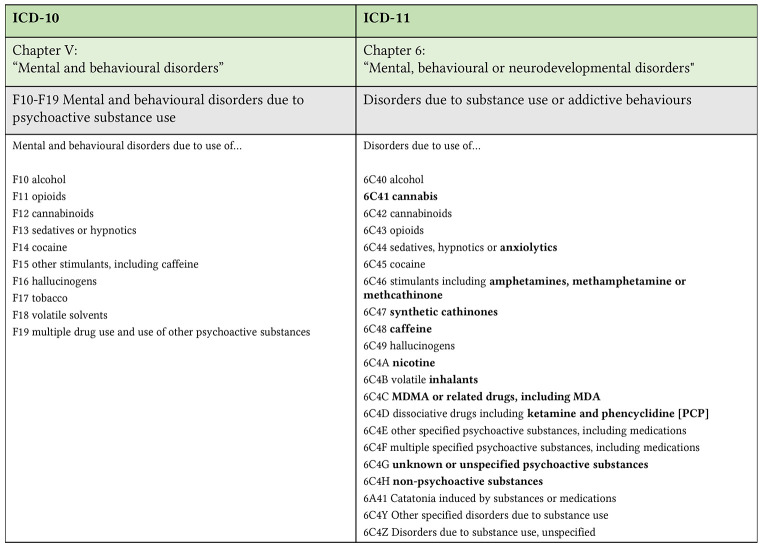
List of Substances in ICD-10 and ICD-11 *Note.* Entries in bold show the new or differently classified/named substances in ICD-11 compared to ICD-10.

Disorders due to substance use include disorders that result from a single occasion or repeated use of substances that have psychoactive properties, including certain medications, and are classified according to the substance. The list of substances has been broadened from 9 (ICD-10) to 14, to comprehend contemporary patterns of use: alcohol, cannabis, synthetic cannabinoids, opioids, sedative hypnotics and anxiolytics, cocaine, stimulants including amphetamine methamphetamine or methcathinone, synthetic cathinones, caffeine, hallucinogens, nicotine, volatile inhalants, MDMA and related drugs, dissociative drugs including ketamine and phencyclidine (Poznyak et al., 2018). Other classes have been added to include for those substances that are not mentioned and are known of not known: “Disorders due to use of…” other specified psychoactive substances, including medications; multiple specified psychoactive substances, including medications; unknown or unspecified psychoactive substances; non-psychoactive substances. Figure 2 illustrates the differences between the list of substances in ICD-10 and ICD-11. The structure of the classification implies that diagnosis should start from the substance rather than the clinical syndrome. The grouping revision is meant to allow capturing health information to be used in different contexts, support accurate monitoring and inform prevention and treatment. Following the list of substance classes is the list of specific diagnostic categories that apply to the classes of psychoactive substances (Reed et al., 2019; World Health Organization, 2019).

#### Substance Use Related Diagnoses: Innovations in ICD-11

Introduction of single episodes of substance use and of harmful patterns of substance use are among the main features introduced in this version of the ICD for classification of primary diagnoses of substance use disorders (SUD): while with the ICD-10 these were only “Substance dependence” and “Harmful substance use” classifications, with the ICD-11 the primary diagnoses classes are “Substance dependence”, “Harmful Pattern of Psychoactive Substance Use” and “Episode of Harmful Psychoactive Substance Use”. One of these three diagnoses, or “Disorder due to substance use, unspecified” – when the use pattern in unknown at the time of evaluation – must be given when making a diagnosis of a disorder due to substance use (World Health Organization, 2019). These categories are hierarchical and mutually exclusive, in such a way that only one of these can diagnosed for one substance group, therefore removing overlapping and ambiguity.

Early identification and response of SUD can be eased from having different categories for harmful substance use and substance dependence as these can be addressed with different intervention schemes, for instance there are substance use patterns that may benefit from brief psychological interventions (for instance motivational interviewing), while other require more extensive treatment (such as detoxification or agonist maintenance). In addition, the WHO considers harmful consumption categories to be very important for understanding the impact of substance use on public health in morbidity and mortality statistics (First et al., 2021).

Furthermore, there are a number of diagnoses that can be added to the primary ones, which include “Substance intoxication”, “Substance withdrawal” and different “Substance induced mental disorders”. The manual includes also categories related to “Hazardous substance use”, which are classified in Chapter 24, ‘Factors Influencing Health Status or Contact with Health Services’, and not considered to be mental disorders and can be referred to in cases where no evident harm has occurred but the pattern of use increases the risk of harmful health consequences to the user, or to others, in a way that advice from health professionals is needed (Reed et al., 2019; World Health Organization, 2019).

#### Episode of Harmful Psychoactive Substance Use

Inclusion of the single episode of harmful substance use in the ICD-11 is noteworthy, as it allows for early intervention and prevention of increased use and worsening of the condition and harm. The diagnosis should follow an episode where damage has been caused to someone’s physical or mental health, not only referred to the user but also to others: this is an important added value of the ICD-11, where harm to the health of others is explicitly included (Reed et al., 2019). The episode of harmful use usually refers to acute effects and may include substance-induced psychological disorders and should not include harm due to a known harmful pattern of use (World Health Organization, 2019).

#### Harmful Pattern of Psychoactive Substance Use

The harmful pattern of use definition, instead, indicates a case where interventions must be intensified, and refers to a situation where clinically significant harm to a person’s physical or mental health is evident, and can be due not only to the direct intoxicating effects of the substance, but also to secondary effects or harmful route of administration. The pattern can be further specified as episodic or continuous and should be detected for a period of at least one year for episodic use and at least one month for continuous use. Furthermore, harm to health should not be better accounted for by another medical condition or another mental disorder, including another disorder due to substance use, such as substance withdrawal or substance dependence. Harm caused by substance dependence can be similar to that observed in harmful pattern of psychoactive substance use, however, alcohol dependence also includes additional features of the diagnosis and requires at least two of three central features to be present at the same time: impaired control after substance use, substance use becomes an increasing priority in life, physiological features that indicate neuroadaptation to the substance, such a tolerance and withdrawal symptoms (World Health Organization, 2019).

#### Severity Qualifiers for Substance Intoxication

Diagnosis of substance intoxication requires some essential characteristics, that include transient and clinically significant alteration – such as in behavior, consciousness or coordination – that appear during or shortly after substance use, the pharmacological effects of which must be compatible with the symptoms. Intoxication can last from only a few minutes or even several days after the episode of use. The effects of intoxication are limited in time and fade away as the substance is cleared from the body and symptoms are not better attributable to other medical conditions or mental disorders. The ICD-11 allows for specification of severity of intoxication, that can be classified as mild, moderate or severe, and depends on a variety of factors, such as the amount of substance used, its half-life and the route of administration, and of course individual susceptibility which can be influenced from body weight, tolerance or concurrent conditions such as kidney of liver impairment. Substance intoxication is considered mild if disturbances in psychophysiological functions and responses (for instance attention, judgement or motor coordination) are clinically recognizable but there is no – or little – disturbance in the level of consciousness. In moderate intoxication, instead, the above-mentioned disturbances are evident and the tasks that require psychophysiological functioning and response are substantially impaired. There is also some disturbance in the level of consciousness.

Severe substance intoxication is a state in which motor coordination, attention and judgement are obviously impaired, as well as the level of consciousness. The person may not be capable of self-care or self-protection and may not be capable to communicate or cooperate with assessment and intervention. The intensity of intoxication decreases after reaching a peak of absorption of the substance, and the effects eventually disappear in there is no further use of the substance (World Health Organization, 2019).

### Disorders Due to Addictive Behaviors

The new section introduced in the 6^th^ ICD-11 chapter, called “Disorders due to addictive behaviors”, includes “Gambling disorder”, which was previously listed in the category “Habit and impulse control disorders (ICD-10)”, and the new diagnostic category “Gaming disorder” (Saunders, 2017). Diagnosis of gambling and gaming disorders need the manifestation of clinical signs and functional impairment that are observed for a period of at least 12 months, unless severe symptoms arise. Both gambling and gaming disorders are classified as “predominantly online” or “predominantly offline” and are characterized by a pattern of persistent or recurrent behavior. The disorders are defined by impaired control over gambling or gaming, increasing priority given to it, and continuation or escalation despite the occurrence of negative consequences. The pattern of the behavior may be continuous or episodic and recurrent, and results in marked distress or significant impairment in important areas of functioning, such as occupational, family and social life.

#### Gambling Disorder

In ICD-10 gambling was classified under the “Disorders of adult personality and behavior” section “Habit and impulse disorders” and was named “Pathological gambling”. Since recent evidence shows important phenomenological analogies between substance use disorders and disorders due to addictive behaviors, gambling has been associated, together with gaming, in the “Disorders due to Substance Use and Addictive Behaviors” section. This change is important also because a high co-occurrence has been detected within the phenomena, as well as the fact that they are both initially pleasurable and then followed by progression to loss of hedonic value and need for increased use. There is also some scientific evidence that disorders due to substance use and disorders due to addictive behaviors share similar neurobiology, especially activation and neuroadaptation within the reward and motivation neural circuits (Fauth-Bühler et al., 2017; Reed et al., 2019).

#### Gaming Disorder

Gaming disorder, either ‘digital gaming’ or ‘video-gaming’, is described as pattern of persistent or recurrent gaming behavior, which may be online or offline, characterized by impaired control over gaming in terms onset, frequency, intensity, duration, termination, and context. Furthermore, increasing priority is given to gaming in such a way that it takes precedence over other life interests and daily activities and, despite the occurrence of negative consequences, the disorder shows continuation or escalation of gaming (World Health Organization, 2019). Solid evidence and intensive discussions among experts over the past years recognized excessive gaming patterns as a clinically significant syndrome, leading to the inclusion of gaming disorder in the 11^th^ revision of the ICD, making a diagnosis for this disfunction a real possibility for patients and clinicians, where the issue is of such a nature and intensity that it results in marked distress or significant impairment in personal, family, social, educational or occupational functioning (Borges et al., 2021; World Health Organization, 2018). In fact, implications of gaming disfunction are not limited to gaming itself, but come along with other health issues, such as aggressive behaviors, depression, insufficient physical activity, unhealthy diet, eyesight and hearing issues and sleep deprivation (Higuchi et al., 2021; World Health Organization, 2018). Unlike gambling disorder, gaming disorder does not involve the betting of money or other valuables with the hope of obtaining something of greater value. If gaming behavior is focused on wagers (for instance internet poker), gambling disorder is generally the more appropriate diagnosis (World Health Organization, 2019).

## ICD-11 and DSM-5

The ICD and the DSM both have a substantial impact of psychiatric practice and research worldwide, and much effort has been made over the years to harmonize the two classifications and both the WHO and the American Psychiatric Association believe that the differences between the two systems should be minimized and maintained only if conceptually justified (First et al., 2021; Reed et al., 2019). Nevertheless, there are some significant differences in the classification of SUD between the ICD-11 and the DSM-5.

The ICD-11 paragraph “Disorders due to substance use and addictive behaviors” has a corresponding one in the DSM-5: “Substance-related and addictive disorders”. In order to facilitate data collection on their public health impact, some psychoactive substances have been added in the ICD-11 due to their increasing global importance (European Monitoring Centre for Drugs and Drug Addiction and Eurojust, 2016): synthetic cannabinoids (in the DSM‐5 are included in the cannabis class), cocaine (in the DSM‐5 are included in the stimulant class), synthetic cathinones (in the DSM‐5 included in the “other or unknown” class), and methylenedioxyphenethylamine (MDMA) (in the DSM‐5 are included in the hallucinogen class) (First et al., 2021).

Distinct categories for pattern of use included in the ICD-11 are discussed above, the DSM-5, instead, considers only one “Substance use disorder” category, and identifies three levels of severity depending on the number of recognized symptoms among a list of 11: two or three symptoms identify mild SUD, four or five symptoms identify moderate SUD, and six or more symptoms identify severe SUD. Furthermore, DSM-5 does not consider classification of SUD based on harm caused to the person’s physical or mental health or health of others.

Although there is a noticeable similarity between the DSM-5 11 classifications for SUD and the three ICD-11 categories, a number of cases detected with DSM-5 would not find correspondence in the ICD-11: diagnosis of SUD in ICD-11 requires two out of three items, while in DSM-5 two out of 11. “Craving” and “Recurrent use in situations which are physically hazardous” are two items of DSM-5 that are not included nor have a correspondence in ICD-11. Furthermore, all the items related to a substance taking over in daily life activities described in the DSM-5: time spent using or obtaining substances, failure to fulfill role obligations, continued use despite social or interpersonal problems, important activities given up, and continued use despite physical or psychological problems, in ICD-11 are represented in only one category: “increasing precedence of substance use over other aspects of life” (First et al., 2021). All the above might imply that, since there is not a complete homogeneity between the two tools in identifying all the SUD categories, different diagnoses can be made for some groups of SUD (Degenhardt et al., 2019).

As for gaming disorder, some studies suggest that there might be noticeable differences between the two classification systems in gaming disorder cases detection, where prevalence of cases detected with the DSM-5 are much higher compared to ICD-11 (Borges et al., 2021). However, clinical validity studies are needed in order to assess these differences.

## Conclusions

Overall, ICD-11 can represent a new opportunity for several harmful behaviors and for those who are in need for treatment to be timely identified, filling the existing therapeutic gap and increasing the coverage of alcohol and substance use disorders. ICD-11 also pushes for some needed changes, particularly in the post-COVID era (López-Pelayo et al., 2020), to support a much more integrated approach aimed at using standard tools to identify the level of risk as well as training on how to ensure an adequate form of intervention valuing renewed treatment systems for substance use disorders. Finally, the new definitions adopted by ICD-11 call for renewed public health response and policies fostering the multi-professional and multidisciplinary management of alcohol and substance abuse treatment, giving to these forms of addiction new chances also towards the reaching of the UN 2030 Agenda Sustainable Development Goals (SDGs) (United Nations, 2015), aimed at ensuring healthy lives and promote well-being for all ages by mean "strengthen the prevention and treatment of substance abuse including narcotic drug abuse and harmful use of alcohol".
